# Supplementary material for: The biogeography of bent-toed geckos, Cyrtodactylus (Squamata: Gekkonidae)
Source: PeerJ. 2022 Mar 22;10:e13153. doi: 10.7717/peerj.13153 (PMC8953499; doi:10.7717/peerj.13153)
Supplement: Table S1 — Species can be cross-referenced to Figure 3 by GenBank accession. [file peerj-10-13153-s001.docx]

| **Supplementary Table S1.** Species, habitat preference with supporting references, species group designations, and GenBank accession numbers for specimens used in the phylogenetic analysis. Species can be cross-referenced to Figure 3 by their GenBank no. | | | | |
| --- | --- | --- | --- | --- |
| **species** | **habitat** | **reference** | **species group** | **GenBank no.** |
| *Cyrtodactylus adorus* | granite | Shea et al. [1] | *tuberculatus* | KT363927 |
| *C. aequalis* | granite | Grismer et al. [2] | *sinyineensis* | MF872275 |
| *C.* cf. *agamensis* | general | herein | *agamensis* | MH248907 |
| *C. agusanensis* | general | Welton et al. [3] | *philippinicus* | HQ154532 |
| *C. albofasciatus* | terrestrial | Agarwal & Karanth [4] | *triedrus* | KM878625 |
| *C. cf. albofasciatus* | terrestrial | herein | *triedrus* | JX440521 |
| *C. amphipetraeus* | general | Chomdej et al. [5] | *sinyineensis* | MT550630 |
| *C. angularis* | karst | Nabhitabhata and Chan-ard [6] | *angularis* | GU550717 |
| *C. annandalei* | terrestrial | Field notes at California Academy of Sciences | *peguensis* | JX440524 |
| *C. annulatus* | general | Brown and Rico [7]; Welton et al. [8] | *philippinicus* | JX678807 |
| *C.* cf. *annulatus* | general | herein | *philippinicus* | GU366085 |
| *C. arcanus* | general | Oliver et al. [9] | *arcanus* | JQ820314 |
| *C. astrum* | karst | Grismer et al. [10] | *pulchellus* | JX519472 |
| *C. atremus* | trunk | Kraus and Weijola [11] | *sermowaiensis* | MN640774 |
| *C. aunglini* | karst | Grismer et al. [12] | *khasiensis* | MH764589 |
| *C. auralensis* | general | Murdoch et al. [13] | *intermedius* | KT013127 |
| *C. aurensis* | general | Grismer [14] | *philippinicus* | JX440525 |
| *C. auribalteatus* | karst | Ellis and Pauwels [15]; Sumontha et al. [16] | *chauquangensis* | AP018116 |
| *C. australotitiwangsaensis* | granite | Grismer et. al. [10] | *pulchellus* | JX519484 |
| *C. ayeyarwadyensis* | general | Mahony et al. [17] | *khasiensis* | JX440526 |
| *C. badenensis* | cave | Nguyen et al. [18] | orphan | MT953468 |
| *C. baluensis* | trunk | Hikida [19] | *philippinicus* | GU366079 |
| *C. bansocensis* | karst | Luu et al. [20] | *angularis* | MT953469 |
| *C. battalensis* | general | Khan [21] | *lawderanus* | KC151984 |
| *C.* cf. *battalensis* | general | here | *lawderanus* | KC151983 |
| *C. batucolus* | granite | Grismer et al. [22] | *darmandvillei* | JQ889178 |
| *C. bayinnyiensis* | karst | Grismer et al. [23] | *sinyineensis* | MH198647 |
| *C. bichnganae* | karst | Ngo and Grismer [24] | *chauquangensis* | MF169953 |
| *C. bidoupimontis* | trunk | Nazarov et. al. [25] | *irregularis* | MT953470 |
| *C. bintangrendah* | granite | Grismer et al. [10] | *pulchellus* | JX519487 |
| *C. bintangtinggi* | granite | Grismer et al. [10] | *pulchellus* | JX519494 |
| *C. biordinis* | arboreal | Brown and McCoy [26] | orphan | MF673951 |
| *C. borbrovi* | karst | Nguyen et al. [27] | *chauquangensis* | MT953471 |
| *C. bokorensis* | general | Murdoch et al. [13] | *intermedius* | KT013147 |
| *C. boreoclivus* | trunk | Oliver et al. [28]; herein | *capreoloides* | JQ820307 |
| *C. brevidactylus* | terrestrial | Field notes at California Academy of Sciences; Myint Kyaw Thura. Personal communication, 2019 | *khasiensis* | JX440527 |
| *C. brevipalmatus* | arboreal | Grismer [29–30] | *brevipalmatus* | MT953472 |
| *C.* cf. *brevipalmatus* | arboreal | herein | *brevipalmatus* | MT468899 |
| *C.* cf. *brevipalmatus* | arboreal | herein | *brevipalmatus* | USMHC 2555 |
| *C. bugiamapensis* | general | Nazarov et al. [25] | *irregularis* | MT953473 |
| *C. calamei* | karst | Luu et al. [20] | *angularis* | MT953474 |
| *C. caovansungi* | general | Orlov et al. [31] | *irregularis* | MF169954 |
| *C. capreoloides* | general | Oliver et al. [9]; herein | *capreoloides* | JQ820310 |
| *C. cardamomensis* | general | Murdoch et al. [13] | *intermedius* | KT013115 |
| *C. cattienensis* | general | Geissler et al. [32] | *irregularis* | MF169956 |
| *C.* cf. *cattienensis* | general | B. L. Stuart. Personal communication, 2019 | *irregularis* | MW713947 |
| *C.* cf. *cattienensis* | general | Ostrowski et al. [37] | *irregularis* | MW713948 |
| *C. cavernicolus* | karst | Hikida [19] | *philippinicus* | JX440528 |
| *C. cayuensis* | general | Che et al. [33] | *khasiensis* | MW792058 |
| *C. chamba* | granite | Agarwal et al. [34] | *lawderanus* | KM255191 |
| *C. chanhomeae* | karst | Ellis and Pauwels [15]; Bauer et al. [35] | *angularis* | JX440529 |
| *C. chaunghanakwaensis* | karst | Grismer et al. [2] | *sinyineensis* | MH198644 |
| *C. chauquangensis* | karst | Quang et al. [36] | *chauquangensis* | MT953475 |
| *C. chrysopylos* | karst | Grismer et al. [12] | *khasiensis* | MH764601 |
| *C. collegalensis* | terrestrial | Mirza et al. [38]; Agarwal and Karanth [4] | *triedrus* | KX632365 |
| *C. condorensis* | granite | Grismer and Grismer [39] | *condorensis* | KT013196 |
| *C.* cf. *consobrinus* | trunk | Hikida [19]; Grismer [30] | *malayanus* | GU550725 |
| *C.* cf. *consobrinus* | trunk | herein | *malayanus* | JX440528 |
| *C. crustulus* | general | Oliver et al. [40] | *sermowaiensis* | MN640776 |
| *C. cryptus* | general | Heidrich et al. [41] | *irregularis* | MT953476 |
| *C. cucdongensis* | granite | Schneider et al. [42] | *irregularis* | MF169959 |
| *C. cucphuongensis* | karst | Ngo and Chan [43] | *chauquangensis* | MT953477 |
| *C. culaochamensis* | granite | Ngo et al. [44] | *irregularis* | KT013199 |
| *C. dammathetensis* | karst | Grismer et al. [2] | *sinyineensis* | MF872277 |
| *C. darevskii* | karst | Nazarov et al. [45] | *angularis* | MT953478 |
| *C. darmandvillei* | general | Auffenberg [46] | *darmandvillei* | KU232615 |
| *C.* cf. *darmandvillei* | general | Riyanto et al. [47] | *darmandvillei* | KU232616 |
| *C.* cf. *darmandvillei* | general | Riyanto et al. [47] | *darmandvillei* | KU232618 |
| *C.* cf. *darmandvillei* | general | Riyanto et al. [47] | *darmandvillei* | KU232617 |
| *C.* cf. *darmandvillei* | general | Riyanto et al. [47] | *darmandvillei* | KU232623 |
| *C. dati* | general | Ngo [48] | *irregularis* | KT013104 |
| *C. dattkyaik* | karst | Grismer et al. [49] | *sinyineensis* | MN534902 |
| *C. dayangbuntingensis* | karst | Quah et al. [50] | *pulchellus* | MN125090 |
| *C. deccanensis* | terrestrial | Agarwal and Karanth [4] | *triedrus* | KM878615 |
| *C.* cf. *deccanensis* | terrestrial | herein | *triedrus* | KM878628 |
| *C. doisuthep* | granite | Kunya et al. [51] | *chauquangensis* | MT550626 |
| *C. dumnuii* | karst | Bauer et al. [52] | *chauquangensis* | MW713972 |
| *C. durio* | arboreal | Grismer et. al. [53] | *lateralis* | KU893159 |
| *C. eisenmanae* | cave | Ngo and Bauer [54] | *condorensis* | JX440534 |
| *C. elok* | arboreal | Grismer [29–30]; Dring [55] | *lateralis* | JQ889180 |
| *C. epiroticus* | trunk | Kraus [56] | *louisiadensis* | JX440535 |
| *C. equestris* | trunk | Oliver et al. [57]; herein | *novaeguineae* | KT835458 |
| *C. erythrops* | karst | Bauer et al. [58] | *chauquangensis* | MW713958 |
| *C. evanquahi* | karst | Wood et al. [59] | *pulchellus* | MN586889 |
| *C. fasciolatus* | general | Husain and Ray [60] | *fasciolatus* | KM255184 |
| *C.* cf. *fasciolatus* | general | I. Agarawal. Personal communication, 2019 | *fasciolatus* | KM255185 |
| *C. fraenatus* | general | Batuwita and Bahir [61] | *triedrus* | MW713940 |
| *C. gansi* | general | Field notes at California Academy of Sciences | *khasiensis* | JX440537 |
| *C. gialaiensis* | general | Luu et al. [62] | *irregularis* | MT953479 |
| *C. grismeri* | cave | Ngo and Bauer [54] | *condorensis* | JX440538 |
| *C. guakanthanensis* | karst | Grismer et al. [63] | *sworderi* | KU253576 |
| *C. gubaot* | general | Welton et al. [64] | *philippinicus* | KU309336 |
| *C. gubernatoris* | general | Annandale [65] | *peguensis* | KM255204 |
| *C. gunungsenyumensis* | karst | Grismer et al. [66] | *sworderi* | KU253584 |
| *C. guwahatiensis* | general | Agarwal et al. [67] | *khasiensis* | KM255194 |
| *C. halbeii* | general | Mirza et al. [68] | *khasiensis* | MT341524 |
| *C.* cf. *halbeii* | general | I. Agarwal (Personal Commmunication 2019) | *khasiensis* | KM255193 |
| *C. hidupselamanya* | karst | Grismer et al. [69] | *pulchellus* | KX011420 |
| *C. himalayanus* | general | Duda and Sahi [70]; I. Agarwal. Personal communication, 2019 | *lawderanus* | KM255187 |
| *C. hinnamnoensis* | karst | Luu et al. [20] | *angularis* | MT953480 |
| *C. hontreensis* | cave | Ngo et al. [71] | *intermedius* | JX440539 |
| *C. hoskini* | granite | Shea et al. [1]; Reidel et al. [72] | *tuberculatus* | KT363931 |
| *C. houaphanensis* | karst | Schneider et al. [73] | *chauquangensis* | MW792067 |
| *C. huongsonensis* | karst | Luu et al. [74] | *chauquangensis* | MT953481 |
| *C. huynhi* | granite | Ngo and Bauer [75] | *irregularis* | MF169963 |
| *C. ingeri* | trunk | Hikida [19] | *philippinicus* | MN884158 |
| *C. interdigitalis* | arboreal | Ellis and Pauwels [15]; Ulber [76]; Nabhitabhata and Chan-ard [6]; Luu et al [20] | *brevipalmatus* | MW792061 |
| *C.* cf. *interdigitalis* | arboreal | herein | *brevipalmatus* | MT468902 |
| *C.* cf. *interdigitalis* | arboreal | herein | *brevipalmatus* | MT468909 |
| *C.* cf. *interdigitalis* | arboreal | herein | *brevipalmatus* | JQ889181 |
| *C. intermedius* | general | Murdoch et al. [13] | *intermedius* | KT013138 |
| *C.* cf. *intermedius* 1 | general | herein | *intermedius* | GU550210 |
| *C.* cf. *intermedius* 2 | general | herein | *intermedius* | KT013117 |
| *C.* cf. *intermedius* 3 | general | herein | *intermedius* | JX519469 |
| *C. inthanon* | granite | Kunya et al. [77] | *sinyineensis* | MT550625 |
| *C. irregularis* | general | Nazarov et al. [78, 25] | *irregularis* | JX041341 |
| *C. jaegeri* | karst | Luu et al. [79] | *angularis* | MT953482 |
| *C. jaintiaensis* | general | Agarwal et al. [67] | *khasiensis* | KM255195 |
| *C. jambangan* | general | Welton et al. [64] | *philippinicus* | GU366102 |
| *C. jarakensis* | general | Grismer et al. [22] | *agamensis* | MF169965 |
| *C. jarujini* | karst | Ellis and Pauwels [15]; Sumontha et al. [80] | *angularis* | JX440541 |
| *C. jelawangensis* | granite | Grismer et al. [81] | *pulchellus* | KJ659852 |
| *C. jellesmae* | general | Hayden et al. [82] | *darmandvillei* | JX440542 |
| *C. jeyporensis* | terrestrial | Agarwal and Karanth [4] | *triedrus* | KM878616 |
| *C. kazirangaensis* | general | Agarwal et al. [67] | *khasiensis* | KM255170 |
| *C. khasiensis* | general | Li [83] | *khasiensis* | KM255188 |
| *C. kimberleyensis* | general | Bauer and Doughty [84] | *darmandvillei* | JX440544 |
| *C. kingsadai* | granite | Ziegler et al. [85] | *irregularis* | MT953483 |
| *C. klugei* | trunk | herein | *louisiadensis* | HQ401197 |
| *C. kohrongensis* | general | Grismer et al. [86] | *intermedius* | KT013124 |
| *C. kulenensis* | sandstone | Geissler et al. [87] | *intermedius* | MW717585 |
| *C. laangensis* | karst | Murdoch et al. [13] | *intermedius* | KT013158 |
| *C. langkawiensis* | karst | Grismer et al. [10] | *pulchellus* | JX519502 |
| *C. lateralis* | arboreal | Harvey et al. [88] | *lateralis* | KU893162 |
| *C. lawderanus* | terrestrial | I. Agarwal. Personal communication, 2019 | *lawderanus* | KM255190 |
| *C. leegrismeri* | general | Chan and Norhayati [89] | *condorensis* | KT013201 |
| *C. lekaguli* | karst | Grismer et al. [10] | *pulchellus* | KX011425 |
| *C. lenggongensis* | karst | Grismer et al. [69] | *pulchellus* | JX519488 |
| *C. lenya* | karst | Connette et al. [90] | *oldhami* | KY041653 |
| *C. limajalur* | karst | Davis et al. [91] | *malayanus* | MK477177 |
| *C. linnoensis* | karst | Grismer et al. [2] | *yathepyanensis* | MF872295 |
| *C. linnwayensis* | karst | Grismer et al. [2] | *linnwayensis* | MF872288 |
| *C. lomyenensis* | granite | Ngo and Pauwels [92] | *angularis* | MF169966 |
| *C.* cf. *loriae* Wau | trunk | dan Mulyadi [93] Oliver et al. [28] | *loriae* | JQ820299 |
| *C.* cf. *loriae* Bunisi | trunk | herein | *loriae* | HQ401209 |
| *C.* cf. *loriae* Fane | trunk | herein | *loriae* | MW713976 |
| *C.* cf. *loriae* Yuro | trunk | herein | *loriae* | MW713975 |
| *C. louisiadensis* | trunk | Kraus [56]; herein | *louisiadensis* | HQ401190 |
| *C. macrotuberculatus* | general | Grismer and Norhayti [94] | *pulchellus* | JX519519 |
| *C. maelanoi* | general | Grismer et al. [95] | *sinyineensis* | MT823267 |
| *C. majulah* | swamp | Grismer et al. [96] | *agamensis* | JX988529 |
| *C. malayanus* | trunk | Hikida [19]; Grismer [30] | *malayanus* | GU550733 |
| *C.* cf. *malayanus* | trunk | herein | *malayanus* | MK477176 |
| *C.* cf. *malayanus* | trunk | herein | *malayanus* | MK477159 |
| *C.* cf. *malayanus* | trunk | herein | *malayanus* | MK477180 |
| *C. mamanwa* | general | herein | *philippinicus* | GU550820 |
| *C. manos* | general | Oliver et al. [9] | *arcanus* | JQ820322 |
| *C. marmoratus* | general | dan Mulyadi [93] | *marmoratus* | JX440546 |
| *C.* cf. *matsuii* | trunk | Davis et al. [97] | *philippinicus* | MN884159 |
| *C. mcdonaldi* | karst | Shea et al. [1] | *tuberculatus* | HQ401139 |
| *C. medioclivus* | general | Oliver et al. [9]; herein | *capreoloides* | JQ820294 |
| *C. meersi* | terrestrial | Grismer et al. [98] | *peguensis* | MH624104 |
| *C. metropolis* | karst | Grismer et al. [99] | *agamensis* | KU253578 |
| *C. mimikanus* | general | Brown and Parker [100]; herein | *novaeguineae* | JQ820316 |
| *C. minor* | general | Oliver et al. [9] | *capreoloides* | JQ820318 |
| *C. mombergi* | general | Grismer et al. [101] | *khasiensis* | MN059869 |
| *C. montanus* | general | Agarwal et al. [67] | *khasiensis* | KM255200 |
| *C. muanfuangensis* | karst | Sitthivong et al. [102] | *angularis* | MW792068 |
| *C. multiporus* | karst | Nazarov et al. [45] | *angularis* | MT953484 |
| *C. muluensis* | karst | Davis et al. [91] | *philippinicus* | MK477167 |
| *C. murua* | trunk | Kraus and Allison [103] | *louisiadensis* | KT363953 |
| *C. myaleiktaung* | karst | Grismer et al. [12] | *khasiensis* | MH764589 |
| *C. myintkyawthurai* | karst | Grismer et al. [98] | *peguensis* | MH624111 |
| *C. nagalandensis* | general | Agarwal et al. [67] | *khasiensis* | KM255199 |
| *C. naungkayaingensis* | karst | Grismer et al. [23] | *sinyineensis* | MH198664 |
| *C. nebulosus* | terrestrial | Agarwal and Karanth [4] | *triedrus* | KM878618 |
| *C.* cf. *nebulosus* | terrestrial | herein | *triedrus* | KM878619 |
| *C.* cf. *nebulosus* | terrestrial | herein | *triedrus* | KM878620 |
| *C.* cf. *nebulosus* | terrestrial | herein | *triedrus* | KM878621 |
| *C. ngati* | karst | Le et al. [104] | *brevipalmatus* | MW655790 |
| *C. ngoiensis* | karst | Schneider et al. [73] | *chauquangensis* | MW792066 |
| *C. nigriocularis* | cave | Nguyen et al. [105] | *angularis* | MT953485 |
| *C. novaeguineae* | trunk | Oliver et al. [28] | *novaeguineae* | KT363956 |
| *C. nyinyikyawi* | terrestrial | Grismer et al. [106] | *peguensis* | MH624118 |
| *C. oldhami* | general | Nabhitabhata and Chan-ard [6] | *oldhami* | MF872302 |
| *C.* cf. *oldhami* | general | herein | *oldhami* | AP018118 |
| *C.* cf. *oldhami* | general | herein | *oldhami* | MW713967 |
| *C.* cf. *oldhami* | general | herein | *oldhami* | MT468910 |
| *C.* cf. *oldhami* | general | herein | *oldhami* | MT468911 |
| *C. otai* | karst | Nguyen et al. [107] | *chauquangensis* | MT953486 |
| *C. pageli* | karst | Schneider et al. [108] | *angularis* | MT953487 |
| *C. pantiensis* | swamp | Grismer et al. [22] | *agamensis* | JQ889185 |
| *C. papuensis* | general | Brown and Parker [100] | *marmoratus* | JQ820320 |
| *C. payacola* | swamp | Johnson et al. [109] | *agamensis* | JQ889190 |
| *C. payarhtanensis* | karst | Connette et al. [90] | *oldhami* | KY041654 |
| *C. peguensis* | terrestrial | Grismer et al. [ 106] | *peguensis* | MH756190 |
| *C. petani* | general | Riyanto et al. [110] | *darmandvillei* | KU232620 |
| *C. pharbaungensis* | karst | Grismer et al. [2] | *sadansinensis* | MF872303 |
| *C. philippinicus* | general | Brown et al. [111]; Welton et al. [64] | *philippinicus* | GU550895 |
| *C.* cf. *philippinicus* | general | Brown et al. [111]; Welton et al. [64] | *philippinicus* | GU550825 |
| *C. phnomchiensis* | granite | Neang et al. [112] | *irregularis* | MT066405 |
| *C. phongnhakebangensis* | karst | Loos et al. [113] | *angularis* | MF169970 |
| *C. phumyensis* | granite | Ostrowski et al. [114] | *irregularis* | MW792065 |
| *C. phuocbinhensis* | granite | Nguyen et al. [115] | *irregularis* | MT953488 |
| *C. phuquocensis* | general | Ngo et al. [116] | *intermedius* | MF169971 |
| *C. pinlaungensis* | karst | Grismer et al. [117] | *linnwayensis* | MN030634 |
| *C. pronarus* | granite | Shea et al. [1] | *tuberculatus* | HQ401152 |
| *C. psarops* | general | Harvey et al. [118] | *agamensis* | KR921705 |
| *C. pseudoquadrivirgatus* | general | Rösler et al. [119] | *irregularis* | MF169972 |
| *C. pubisulcus* | general | Hikida [19] | *philippinicus* | JX440551 |
| *C.* cf. *pubisulcus* | general | herein | *philippinicus* | MN884152 |
| *C.* cf. *pubisulcus* | general | herein | *philippinicus* | MK477166 |
| *C. puhuensis* | general | Nguyen et al. [120] | *chauquangensis* | MT953489 |
| *C. pulchellus* | granite | Grismer et al. [10] | *pulchellus* | MF169974 |
| *C. pyadalinensis* | terrestrial | Grismer et al. [106] | *peguensis* | MH624105 |
| *C. pyinyaungensis* | terrestrial | Grismer et al. [2] | *peguensis* | MF872307 |
| *C. quadrivirgatus* | general | Johnson et al. [109]; Grismer [30] | *sworderi* | MF169975 |
| *C. raglai* | cave | Nguyen et al. [121] | *irregularis* | MW675652 |
| *C. ramboda* | trunk | Batuwita and Bahir [61] | *triedrus* | MW713939 |
| *C. redimiculus* | general | Brown and Dimalibot [122] | *philippinicus* | GU550738 |
| *C. rex* | trunk | Oliver et al. [57]; herein | *novaeguineae* | KT835460 |
| *C. rishivalleyensis* | terrestrial | Agarwal [123] | *triedrus* | KX698081 |
| *C. robustus* | trunk | Kraus [56]; herein | *louisiadensis* | JX440554 |
| *C. roesleri* | karst | Ziegler et al. [124] | *angularis* | HLM 0333 |
| *C. rosichonarieforum* | swamp | Riyanto et al. [47] | *agamensis* | KP256188 |
| *C. rubidus* | general | Annandale [65]; Chandramouli [125] | *lateralis* | KM255203 |
| *C. russelli* | general | Grismer et al. [101] | *peguensis* | JX440555 |
| *C. sadanensis* | karst | Grismer et al. [2] | *yathepyanensis* | MF872324 |
| *C. sadansinensis* | karst | Grismer et al. [2] | *sadansinensis* | MF872325 |
| *C. sadleiri* | general | Smith et al. [126] | *darmandvillei* | MH105038 |
| *C. saiyok* | general | Aksornneam et al. [127]; Panitvong et al. [128] | *oldhami* | MF872308 |
| *C.* cf. *saiyok* | general | herein | *oldhami* | MW713964 |
| *C. salomonensis* | trunk | Rösler et al. [129] | *louisiadensis* | JX440556 |
| *C. sangi* | general | Pauwels et al. [130] | *irregularis* | MW713956 |
| *C. sanook* | karst | Pauwels et al. [131] | *oldhami* | MW713973 |
| *C. sanpelensis* | karst | Grismer et al. [2] | *sadansinensis* | MF872345 |
| *C. semenanjungensis* | swamp | Grismer and Leong [132] | *agamensis* | JQ889177 |
| *C. semicinctus* | general | Harvey et al. [118] | *agamensis* | KR921713 |
| *C. septentrionalis* | general | Agarwal et al. [67] | *khasiensis* | MH971164 |
| *C. septimontium* | granite | Murdoch et al. [13] | *intermedius* | MH940233 |
| *C. seribuatensis* | intertidal | Youmans and Grismer [133] | *darmandvillei* | JX440557 |
| *C. sermowaiensis* | general | Rösler et al. [129]; Kraus and Weijola [11] | *sermowaiensis* | JQ820296 |
| *C. serratus* | trunk | herein | *loriae* | JQ820297 |
| *C.* sp. West Sepik | trunk | herein | *loriae* | MW713977 |
| *C. sharkari* | karst | Grismer et al. [81] | *pulchellus* | KJ659853 |
| *C. shwetaungorum* | karst | Grismer et al. [2] | *linnwayensis* | MF872353 |
| *C. sinyineensis* | karst | Grismer et al. [2] | *sinyineensis* | MF872355 |
| *C. slowinskii* | general | Field notes at California Academy of Sciences | *peguensis* | JX440559 |
| *C. soba* | general | Batuwita and Bahir [61] | *triedrus* | MW713938 |
| *C. sommerladi* | karst | Luu et al. [134] | *angularis* | MT953490 |
| *C. soni* | karst | Le et al. [135] | *chauquangensis* | MT953491 |
| *C. sonlaensis* | karst | Nguyen et al. [136] | *chauquangensis* | MT953492 |
| *C. soudthichaki* | karst | Luu et al. [137] | *angularis* | MT953493 |
| *C.* sp. CDS 2010 | general | herein | *agamensis* | GU550728 |
| *C.* sp. DMSSK 126 | general | herein | *triedrus* | MW792063 |
| *C.* sp. HLM0310 | trunk | herein | *khasiensis* | MW713970 |
| *C.* sp. HLM0313 Kubah | trunk | herein | *philippinicus* | MW792060 |
| *C.* sp. HLM0316 Kon Ka Kinh | trunk | herein | *irregularis* | MW713951 |
| *C.* sp. HLM0324 Lomyen | cave | herein | *angularis* | MW713943 |
| *C.* sp. HLM0349 Nahin | karst | herein | *angularis* | MW713944 |
| *C.* sp. HLM0353 Nahin | general | herein | *angularis* | MW713946 |
| *C.* sp. HLM0354 Kon Tum | general | herein | *irregularis* | MW713955 |
| *C.* sp. HLM0357 Tham Pla | karst | herein | *chauquangensis* | MW713961 |
| *C.* sp. HLM0358 Krabi | General | herein | *oldhami* | MW713969 |
| *C.* sp. HLM0360 Chanthaburi | General | herein | *intermedius* | MW713966 |
| *C.* sp. HLM0362 Khao Yai | General | herein | *intermedius* | MW713965 |
| *C.* sp. HLM0365 Kon Ka Kinh | General | herein | *irregularis* | MW713950 |
| *C.* sp. HLM0366 Chu Mom Ray | General | herein | *irregularis* | MW713954 |
| *C.* sp. HLM0367 Loc Bac | General | herein | *irregularis* | MW713952 |
| *C.* sp. HLM0368 Loc Bao | General | herein | *irregularis* | MW713978 |
| *C.* sp. HLM0371 Ujung Kulon | General | herein | *marmoratus* | MW792056 |
| *C.* sp. HLM0372 Suan Phueng | Arboreal | herein | *brevipalmatus* | MW713960 |
| *C.* sp. IA 2014 | General | I. Agarwal. Personal communication, 2019 | *peguensis* | KM255181 |
| *C.* sp. IA 2014 | General | I. Agarwal. Personal communication, 2019 | *khasiensis* | KM255192 |
| *C.* sp. IA 2014 | Trunk | I. Agarwal. Personal communication, 2019 | *peguensis* | KM255196 |
| *C.* sp. KAC 2015 | General | K. O'Connell. I. Agarwal. Personal communication, 2020 | *marmoratus* | KR921689 |
| *C.* sp. KAC 2015 | General | K. O'Connell. I. Agarwal. Personal communication, 2020 | *marmoratus* | KR921697 |
| *C.* sp. KAC 2015 | General | K. O'Connell. I. Agarwal. Personal communication, 2020 | *marmoratus* | KR921699 |
| *C.* sp. KAC 2015 | General | K. O'Connell. I. Agarwal. Personal communication, 2020 | *marmoratus* | KR921700 |
| *C.* sp. KAC 2015 | General | K. O'Connell. I. Agarwal. Personal communication, 2020 | *agamensis* | KR921711 |
| *C.* sp. KAC 2015 | General | K. O'Connell. I. Agarwal. Personal communication, 2020 | *marmoratus* | KR921720 |
| *C.* sp. Kai Islands | General | Nielson and Oliver [138] | *darmandvillei* | MF706380 |
| *C.* sp. Kirirom | General | herein | *intermedius* | KT013114 |
| *C.* sp. Mamberamo | Trunk | herein | *novaeguineae* | JQ820316 |
| *C.* sp. | General | K. O'Connell. I. Agarwal. Personal communication, 2020 | *agamensis* | MH248914 |
| *C.* sp. Mizoram | General | Agarwal pers. comm., (2019) | *khasiensis* | KM255197 |
| *C.* sp. Mt Pekopekowana | Trunk | Kraus [56]; herein | *louisiadensis* | HQ401193 |
| *C.* sp. Song Thanh | General | herein | *irregularis* | MW713949 |
| *C.* sp. Yingjiang | Karst | herein | *khasiensis* | MW792064 |
| *C.* sp. Timor | General | Nielsen and Oliver [138] | *darmandvillei* | JX440560 |
| *C.* sp. WAMR 109089 | General | Riyanto et al. [110] | *darmandvillei* | KU232625 |
| *C.* sp. WAMR 109893 | General | Riyanto et al. [110] | *darmandvillei* | KU232621 |
| *C.* sp. WAMR 112000 | General | herein | *darmandvillei* | KU232624 |
| *C.* sp. Tak | Granite | herein | *sinyineensis* | MT468903 |
| *C.* sp. Tak | Karst | herein | *sinyineensis* | MT468901 |
| *C.* sp. Kamphaeng Phet | Granite | herein | *sinyineensis* | MT497807 |
| *C.* sp. Tham | Granite | herein | *chauquangensis* | MT468908 |
| *C. speciosus* | terrestrial | Agarwal and Karanth [4] | *triedrus* | KM878623 |
| *C.* cf. *speciosus* | terrestrial | Mirza et al. [38]; Agarwal and Karanth [4] | *triedrus* | KM878629 |
| *C. spelaeus* | karst | herein | *chauquangensis* | MW713962 |
| *C. spinosus* | arboreal | Linkem et al. [139] | orphan | MT953494 |
| *C. srilekhae* | terrestrial | Agarwal [123] | *triedrus* | KX698084 |
| *C. sumuroi* | general | Welton et al. [3] | *philippinicus* | GU550772 |
| *C. sworderi* | general | Grismer [30] | *sworderi* | JQ889189 |
| *C. takouensis* | granite | Ngo and Bauer [54] | *irregularis* | MF169978 |
| *C. tanim* | karst | Nielsen and Oliver [138] | *capreoloides* | MF706378 |
| *C. taungwineensis* | karst | Grismer et al. [49] | *sinyineensis* | MN534925 |
| *C. tautbatorum* | general | Welton et al. [8] | *philippinicus* | GU550752 |
| *C. taybacensis* | karst | Pham et al. [140] | *chauquangensis* | MT953495 |
| *C. taynguyenensis* | general | Nguyen et al. [115] | *irregularis* | MW792059 |
| *C. tebuensis* | general | Grismer et al. [141] | *sworderi* | JX988528 |
| *C. teyniei* | karst | David et al. [142] | *angularis* | MT953496 |
| *C. thirakhupti* | karst | Pauwels et al. [143]; herein | *oldhami* | AP018115 |
| *C. thylacodactylus* | terrestrial | Murdoch et al. [13]; herein | *intermedius* | KT013163 |
| *C. tibetanus* | general | K. Wang. Personal communication, 2019 | *tibetanus* | YPX1413 |
| *C.* cf. *tibetanus* | general | Shi and Zhoa [144]; K. Wang. Personal communication, 2019 | *tibetanus* | JX440561 |
| *C. tigroides* | karst | Bauer et al. [35]; Aksornneam [127] | orphan | JX440562 |
| *C. timur* | granite | Grismer et al. [81] | *pulchellus* | KJ659857 |
| *C. tiomanensis* | granite | J. Grismer et al. [145] | *agamensis* | GU550734 |
| *C. triedrus* | terrestrial | herein | *triedrus* | MW713937 |
| *C.* cf. *triedrus* | terrestrial | Agarwal and Karanth [4]; Botejue and Wattavidanage [146] | *triedrus* | JX440522 |
| *C.* cf. *triedrus* | terrestrial | herein | *triedrus* | MW713941 |
| *C. trilatofasciatus* | granite | Grismer et al. [10] | *pulchellus* | JX519530 |
| *C. tripartitus* | trunk | Kraus [56] | *louisiadensis* | JQ820317 |
| *C. tripuraensis* | general | I. Agarwal. Personal communication, 2019 | *khasiensis* | KM255183 |
| *C. tuberculatus* | general | Shea et al. [1]; Worthington et al. [147] | *tuberculatus* | KT363943 |
| *C. urbanus* | granite | Purkayastha et al. [148] | *khasiensis* | MN911174 |
| *C. varadgirii* | terrestrial | Agarwal et al. [149] | *triedrus* | KX632368 |
| *C. vilaphongi* | karst | Schneider et. al. [42]; Luu et al. [137] | *chauquangensis* | MT953497 |
| *C. wayakonei* | karst | Nguyen et al. [150]; Yuan and Rao [151]; Luu et al. [137] | *chauquangensis* | MT953498 |
| *C. welpyanensis* | karst | Grismer et al. [2] | *sinyineensis* | MF872360 |
| *C. yakhuna* | terrestrial | Deraniyagala [152] | *triedrus* | MW713942 |
| *C. yangbayensis* | granite | Ngo and Chan [153] | *irregularis* | KT013202 |
| *C.* cf. *yangbayensis* | karst | Ngo and Chan [153] | *irregularis* | MF169968 |
| *C. yathepyanensis* | karst | Grismer et al. [2] | *yathepyanensis* | MF872363 |
| *C. yoshii* | trunk | Hikida [19] | *philippinicus* | JX440565 |
| *C. ywanganensis* | karst | Grismer et al. [154] | *linnwayensis* | MH607610 |
| *C. zebriacus* | general | Taylor [155]; herein | *oldhami* | MW713971 |
| *C. zhaoermi* | general | Shi and Zhao [144] | *tibetanus* | YPX1433 |
| *C. zhenkangensis* | karst | Liu and Rao [156] | *chauquangensis* | MW792062 |
| *C. ziegleri* | general | Nazarov et al. [78] | *irregularis* | HLM0305 |
| *C.* cf. *ziegleri* | general | herein | *irregularis* | HLM0345 |
| *C. zugi* | trunk | Oliver et al. [157] | *novaeguineae* | JQ820306 |

1. Shea, G.; Couper, P.; Wilmer, J.W.; Andrew, A. Revision of the genus *Cyrtodactylus* Gray, 1827 (Squamata: Gekkonidae) in Australia. *Zootaxa* **2011,** *3146*, 1–63. <https://doi.org/10.11646/zootaxa.3146.1.1>

2. Grismer, L.L.; Wood, Jr., P.L.; Myint Kyaw Thura, Thaw Zin, Quah, E.H.S.; Murdoch, M.L.; Grismer, M.S.; Aung Lin, Htet Kyaw, Ngwe Lwin. Twelve new species of *Cyrtodactylus* Gray (Squamata: Gekkonidae) from isolated limestone habitats in east- central and southern Myanmar demonstrate high localized diversity and unprecedented microendemism *Zool. J. Linn. Soc.* **2018,** *182*, 862–959.

<https://doi.org/10.1093/zoolinnean/zlx057>

3. Welton, L.J.; Siler, C.D.; Diesmos, A.; Brown, R.M. Phylogeny-based species delimitation of southern Philippines bent-toed geckos and a new species of *Cyrtodactylus* (Squamata: Gekkonidae) from western Mindanao and the Sulu Archipelago. *Zootaxa* **2010,** *2390*, 49–68. <https://doi.org/10.1093/zoolinnean/zlx057>

4. Agarwal, I.; Karanth, K.P. A phylogeny of the only ground-dwelling radiation of *Cyrtodactylus* (Squamata, Gekkonidae): diversification of *Geckoella* across peninsular India and Sri Lanka. *Mol. Phylo. Evol.* **2015,** *82*, 193–199.

<https://doi.org/10.1016/j.ympev.2014.09.016>

5. Chomdej, S.; Suwannapoom, C.; Pawangkhanant, P.; Pradit, W.; Nazarov, R.A.; Grismer, L.L.; Poyarkov, N.A. A new species *Cyrtodactylus* Gray (Squamata: Gekkonidae) from western Thailand and the phylogenetic placement of *C. inthanon* and *C. doisuthep.* *Zootaxa* **2020,** *4838*, 179–209.

<https://doi.org/10.11646/zootaxa.4838.2.2>

6. Nabhitabhata, J.; Chan-ard, T. *Thailand Red Data: Mammals, Reptiles and Amphibians.* ONEP: Office of Natural Resources and Environmental Policy and Planning, Thailand, 2005, pp. 1–200.

7. Brown, R.; Rico, E. *Cyrtodactylus annulatus.* The IUCN Red List of Threatened Species. Version 2014.2, **2009.** Available at: www.iucnredlist.org (accessed on 14 October 2014).

8. Welton, L.J..; Siler, C.D.; Diesmos, A.; Brown, R.M. A new bent-toed gecko (Genus *Cyrtodactylus*) from Southern Palawan Island, Philippines and clarification of the taxonomic status of *C. annulatus*. *Herpetologica* **2009,** *65*, 328–343.

<https://doi.org/10.1655/08-057r1.1>

9. Oliver, P.M.; Karkkainen, D.T.; Rösler, H.; Richards, S.J. A new species of *Cyrtodactylus* (Squamata: Gekkonidae) from central New Guinea. *Zootaxa*, 2019, *4671*, 119–128.

[https://doi.org/10.1111/j.14636409.2012.00545.x](https://doi.org/10.1111/j.1463-6409.2012.00545.x)

10. Grismer, L.L.; Wood, Jr., P.L.; Quah, E.S.H.; Anuar, S.; Muin, M.A.; Sumontha, M.; Norhayati, A.; Bauer, A.M.; Wangkulangkul, S.; Grismer, J.L.; Pauwels, O.S.G. A phylogeny and taxonomy of the Thai-Malay Peninsula Bent-toed geckos of the *Cyrtodactylus pulchellus* complex (Squamata: Gekkonidae): combined morphological and molecular analyses with descriptions of seven new species. *Zootaxa* **2012,** 3520, 1–55.

<https://doi.org/10.11646/zootaxa.3520.1.1>

11. Kraus, F.; Weijola, V. New species of *Cyrtodactylus* (Squamata: Gekkonidae) from Karkar Island, Papua New Guinea. *Zootaxa* **2019,** *4695*, 529–540.

<https://doi.org/10.11646/zootaxa.1425.1.8>

12. Grismer, L.L.; Wood, Jr., P.L.; Myint Kyaw Thura, Nay Myo Win, Grismer, M.S.; Trueblood, T.A.; Quah, E.S.H. A re-description of *Cyrtodactylus chrysopylos* Bauer (Squamata: Gekkonidae) with comments on the adaptive significance of orange coloration in hatchlings and descriptions of two new species from eastern Myanmar (Burma). *Zootaxa* **2018,** *4527*, 151–185.

<https://doi.org/10.11646/zootaxa.4527.2.1>

13. Murdoch, M.L.; Grismer, L.L.; Wood, Jr., P.L.; Thy, N.; Poyarkov, N.A.; Ngo, V.T.; Nazarov, R.A.; Aowphol, A.; Pauwels, O.S.G.; Nguyen, H.C.; Grismer, J.L. Six new species of the *Cyrtodactylus* *intermedius* complex (Squamata: Gekkonidae) from the Cardamom Mountains and associated highlands of Southeast Asia. *Zootaxa* **2019,** *4554*, 1–62.

<https://doi.org/10.11646/zootaxa.4554.1.1>

14. Grismer, L.L. New species of bent-toed gecko (*Cyrtodactylus* Gray 1827) from Pulau Aur, Johor, West Malaysia. *J. Herpetol.* **2005,** *39*, 424–432.

<https://doi.org/10.1670/3-05a.1>

15. Ellis, M.; Pauwels, O.S.G. The bent-toed geckos (*Cyrtodactylus*) of the caves and karst of Thailand. *Cave and Karst Science* **2012,** *39*, 16–22.

16. Sumontha, M.; Panitvong, N.; Deein, G. *Cyrtodactylus auribalteatus* (Squamata: Gekkonidae), a new cave- dwelling gecko from Phitsanulok Province, Thailand. *Zootaxa* **2010,** *2370*, 53–64.

<https://doi.org/10.11646/zootaxa.2370.1.3>

17. Mahony, S.; Hossain, A.M.; Hossain, M.K.; Kabir, M.M.; Hasan, M.K. *Cyrtodactylus ayeyarwadyensis* Bauer, 2003 (Squamata: Gekkonidae) in Bangladesh with habitat details of new collection localities and a discussion of morphological variation. *Salamandra* **2009,** *45*, 245–250.

18. Nguyen, S.N.; Orlov, N.L.; Darevsky, I.S. Descriptions of two new species of the genus *Cyrtodactylus* Gray, 1827 (Squamata: Sauria: Gekkonidae) from southern Vietnam. *J. Herpetol.* **2006,** *13*, 215–226.

19. Hikida, T. (1990). Bornean gekkonid lizards of the genus *Cyrtodactylus* (Lacertilia: Gekkonidae) with descriptions of three new species. *Japanese J. Herptol.* 13, 91–107.

<https://doi.org/10.5358/hsj1972.13.3_91>

20. Luu, V.Q.; Nguyen, T.Q.; Le, M.D.; Bonkowski, M.; Ziegler. T. A new species of karst-dwelling bent-toed gecko (Squamata: Gekkonidae) from Khammouane Province, central Laos. *Zootaxa* **2016,** *4079*, 87–102.

<https://doi.org/10.11646/zootaxa.4079.1.6>

21. Khan, M.S. A new angular-toed gecko from Pakistan, with remarks on the taxonomy and a key to the species belonging to genus *Cyrtodactylus* (Reptilia: Sauria: Gekkonidae). *Pakistan J. Zool.* **1993,** *25*, 67–73.

22. Grismer, L.L.; Chan, K.O.; Grismer, J.L.; Wood, Jr., P.L.; Belabut, D.M. Three new species of *Cyrtodactylus* (Squamata: Gekkonidae) from Peninsular Malaysia. *Zootaxa* **2008,** *1921*, 1–23.

<https://doi.org/10.11646/zootaxa.1921.1.1>

23. Grismer, L.L.; Wood Jr., P.L.; Myint Kyaw Thura; Quah, E.S.H.; Murdoch, M.L.; Grismer, M.S.; Herr, M.W.; Aung Lin; Htet Kyaw. Three more new species of *Cyrtodactylus* (Squamata: Gekkonidae) from the Salween Basin of eastern Myanmar underscore the urgent need for the conservation of karst habitats. *J. Natur. Hist.*, **2018,** *52*, 1243–1294.

<https://doi.org/10.1080/00222933.2018.1449911>

24. Ngo, V.T.; Grismer, L.L. A new karst dwelling *Cyrtodactylus* (Squamata: Gekkonidae) from Son La province, north-western Vietnam. *Hamadryad* **2010,** *35*, 84–95.

25. Nazarov, R.; Poyarkov, N.A.; Orlov, N.L.; Phung, T.M.; Nguyen, T.T.; Hoang, D.M; Ziegler, T. Two new cryptic species of the *Cyrtodactylus irregularis* complex (Squamata: Gekkonidae) from southern Vietnam. *Zootaxa* **2012,** *3302*, 1–24.

<https://doi.org/10.11646/zootaxa.3302.1.1>

26. Brown, W.C.; McCoy, M. A new species of gecko of the genus *Cyrtodactylus* from Guadalcanal Island, Solomon Islands. *Herpetologica* **1980,** *36*, 66–69.

27. Nguyen, T.Q.; Le, M.D.; Pham, A.V.; Ngo, H.N.; Hoang, C.V;, Pham, C.T.; Ziegler, T. Two new species of *Cyrtodactylus* (Squamata: Gekkonidae) from the karst forest of Hoa Binh Province, Vietnam. *Zootaxa* **2015,** *3985*, 375–390.

<https://doi.org/10.11646/zootaxa.3985.3.3>

28. Oliver, P.; Krey, K.; Mumpuni; Richards, S. A new species of bent-toed gecko (*Cyrtodactylus*, Gekkonidae) from the North Papuan Mountains. *Zootaxa* **2011,** *2930*, 22–32.

<https://doi.org/10.11646/zootaxa.2930.1.2>

29. Grismer, L.L. On the distribution and identification of *Cyrtodactylus brevipalmatus* Smith, 1923, and *Cyrtodactylus elok*, Dring, 1979. *The Raffles Bull. Zool.* **2008,** *56*, 177–179.

<https://doi.org/10.2305/iucn.uk.2018-2.rlts.t101742205a101742208.en>

30. Grismer, L.L. *Lizards of Peninsular Malaysia, Singapore and Their Adjacent Archipelagos*. Edition Chimaira, Frankfürt am Main, Germany; 2011. Pp. 1–728.

31. Orlov, N.L.; Nguyen, T.Q.; Nazarov, R.V.; Anajeva, N.B.; Nguyen, N.S. A new species of the genus *Cyrtodactylus* Gray, 1827 and redescription of *Cyrtodactylus* *paradoxus* (Darevsky et Szczerbak, 1997) [Squamata: Sauria: Gekkonidae] from south Vietnam. Russ. *J. Herpetol.* **2007,** 14, 145–152.

32. Geissler, P.; Nazarov, R.; Orlov, N.L.; Bohme, W.; Phung, T.M.; Nguyen, T.Q.; Ziegler, T. A new species of the *Cyrtodactylus irregularis* complex (Squamata: Gekkonidae) from southern Vietnam. Zootaxa **2009,** *2161*, 20–32.

<https://doi.org/10.11646/zootaxa.2161.1.2>

33. Che, J.; Jiang, K.; Yan, F.; Zhang, Y.-P. *Amphibians and Reptiles of Tibet-Diversity and Evolution*. Science Press, Beijing, China; 2020; pp. 1–867. (In Chinese)

34. Agarwal, I.; Khandekar, A.; Bauer, A.M. A new bent-toed gecko (Squamata: Gekkonidae) from the Western Himalayas, Himachal Pradesh, India *Zootaxa* **2018,** *4446*, 442–454.

<https://doi.org/10.11646/zootaxa.4446.4.2>

35. Bauer, A.M.; Sumontha, M.; Pauwels, O.S.G. Two new species of *Cyrtodactylus* (Reptilia: Squamata: Gekkonidae) from Thailand. *Zootaxa* **2003,** *376*, 1–18.
<https://doi.org/10.11646/zootaxa.376.1.1>

36. Quang, H.X.; Orlov, N.L.; Ananjeva, N.B.; Johns, A.J.; Thao, H.N.; Vinh, D.Q. Description of a new species of the genus *Cyrtodactylus* Gray, 1827 (Squamata: Sauria: Gekkonidae) from the Karst of north central Vietnam. *Russ*. *J. Herpetol.* **2007,** *14*, 98­–106.

37. Ostrowski, S.; Le, M.D.; Ngo, H.T.; Pham, C.T.; Phung, T.M.; Nguyen, T.Q.; Ziegler, T. A new *Cyrtodactylus* (Squamata: Gekkonidae) from Binh Thuan Province, southern Vietnam. *European J. Tax.* **2021,** 731, 47–70.

38. Mirza, Z.A.; Pal, S.; Sanap, R.V. Notes on a ground gecko *Geckoella* cf. *collegalensis* Beddome, 1870 (Squamata, Sauria, Gekkonidae) from India. *Russian J. Herpetol.* **2010,** 17, 8–14.

39. Grismer, L.L.; Grismer, J.L. A re-evaluation of the phylogenetic relationships of the *Cyrtodactylus condorensis* group (Squamata; Gekkonidae) and a suggested protocol for the characterization of rock-dwelling ecomorphology in *Cyrtodactylus*. *Zootaxa*, **2017,** *4300*, 486–504.

<https://doi.org/10.11646/zootaxa.4300.4.2>

40. Oliver, P.M.; Hartman, R.; Turner, C.D.; Wilde, T.A.; Austin, C.C.; Richards, S.J. A new species of *Cyrtodactylus* Gray (Gekkonidae: Squamata) from Manus Island, and extended description and range extension for *Cyrtodactylus sermowaiensis* (De Rooij). *Zootaxa* **2020,** *4728*, 341–356.

<https://doi.org/10.11646/zootaxa.4728.3.3>

41. Heidrich, A.; Rösler, H.; Than, V.N.; Böhme, W.; Ziegler, T. Another new *Cyrtodactylus* (Squamata: Gekkonidae) from Phong Nha-Ke Bang National Park, central Truong Son, Vietnam. *Zootaxa* **2007,** *1445*, 35–38.

<https://doi.org/10.11646/zootaxa.1445.1.3>

42. Schneider, N.; Nguyen, T.Q.; Le, M.D.; Nophaseud, L.; Bonkowski, M.; Ziegler, T. A new species of *Cyrtodactylus* (Squamata: Gekkonidae) from the karst forest of northern Laos. *Zootaxa* **2014,** *3835*, 80­–96.

<https://doi.org/10.11646/zootaxa.3835.1.4>

43. Ngo, V.T.; Chan, K.O. A new karstic cave-dwelling *Cyrtodactylus* Gray (Squamata: Gekkonidae) from Northern Vietnam. *Zootaxa* **2011,** *3125*, 51–63.

44. Ngo, T.V.; Grismer, L.L.; Thai, P.H.; Wood, Jr., P.L. A new endemic insular Bent-toed Gecko (Squamata: Gekkonidae: *Cyrtodactylus*) from Quang Nam Province, Central Vietnam. *Zootaxa* **2020,** *4766*, 389-400.

<https://doi.org/10.11646/zootaxa.4766.2.7>

45. Nazarov, R.; Poyarkov, N.A.; Orlov, N.A.; Nguyen, N.S.; Mito, K.D.; Martynov, A.A.; Konstantinov, E.L.; Chulisov, A.S. A review of genus *Cyrtodactylus* (Reptilia: Sauria: Gekkonidae) in fauna of Laos with description of four new species. *Proc. Zool. Inst. RAS* **2014,** *318*, 391–423.

46. Auffenberg, W. The herpetofauna of Komodo, with notes on adjacent areas. *Bull. Florida State Mus. Nat. Hist.*, *Biol. Sci.* **1980,** *25*, 39–156.

47. Riyanto, A.; Grismer, L.L.; Wood, Jr., P.L. *Cyrtodactylus rosichonariefi* sp. nov. (Squamata: Gekkonidae), a new swamp-dwelling bent-toed gecko from Bunguran Island (Great Natuna), Indonesia. *Zootaxa* **2015,** *3964*, 114–124.

48. Ngo, V.T. *Cyrtodactylus dati*, a new forest dwelling Bent-toed Gecko (Squamata: Gekkonidae) from southern Vietnam. *Zootaxa*, **2013,** *3616*, 151–64.

<https://doi.org/10.11646/zootaxa.3616.2.4>

49. Grismer, L.L.; Wood, Jr., P.L.; Quah, E.S.H.; Grismer, M.S.; Thura, M.K.; Oaks, J.R.; Lin, A. Two new species of *Cyrtodactylus* Gray, 1827 (Squamata: Gekkonidae) from a karstic archipelago in the Salween Basin of southern Myanmar (Burma). *Zootaxa* **2020,** *4718*, 151–183.

<https://doi.org/10.11646/zootaxa.4718.2.1>

50. Quah, E.S.; Grismer, L.L.; Wood, Jr., P.L.; Shahrul, A. The discovery and description of a new species of Bent-toed Gecko of the *Cyrtodactylus pulchellus* complex (Squamata: Gekkonidae) from the Langkawi Archipelago, Kedah, Peninsular Malaysia. *Zootaxa* **2019,** *4668*, 51–75.

<https://doi.org/10.11646/zootaxa.4668.1.3>.

51. Kunya, K.; Panmongkol, A.; Pauwels, O.S.G.; Sumontha, M.; Meewasana, J.; Bunkhwamdi, W.; Dangsri, S. A new forest-dwelling Bent-toed Gecko (Squamata: Gekkonidae: *Cyrtodactylus*) from Doi Suthep, Chiang Mai Province, northern Thailand. *Zootaxa* **2014,** *3811*, 251–261.

<https://doi.org/10.11646/zootaxa.3811.2.6>

52. Bauer, A.M.; Kunya, K.; Sumontha, M.; Niyomwan, P.; Pauwels, O.S.G.; Chanhome, L.; Kunya, T. *Cyrtodactylus dumnuii* (Squamata: Gekkonidae), a new cave-dwelling gecko from Chiang Mai Province, Thailand. *Zootaxa* **2010,** *2570*, 41–50.

<https://doi.org/10.11646/zootaxa.2570.1.2>

53. Grismer, L.L.; Anuar, S.; Quah, E.S.; Muin, M.A.; Chan, K.O.; Grismer, J.L.; Norhayati, A. A new spiny, prehensile-tailed species of *Cyrtodactylus* (Squamata: Gekkonidae) from Peninsular Malaysia with a preliminary hypothesis of relationships based on morphology. *Zootaxa* **2010,** *2625*, 40–52.

<https://doi.org/10.11646/zootaxa.2625.1.2>

54. Ngo, V.T; Bauer, A.M. Descriptions of two new species of *Cyrtodactylus* Gray, 1827 (Squamata: Gekkonidae) endemic to southern Vietnam. *Zootaxa* **2008,** *1715*, 27–42.

<https://doi.org/10.11646/zootaxa.1715.1.2>

55. Dring, J.C.M. Amphibians and reptiles from northern Trengganu, Malaysia, with descriptions of two new geckos: *Cnemaspis* and *Cyrtodactylus*. *Bull. British Mus. (Nat. Hist.)* **1979,** 34, 181–241.

56. Kraus, F. Taxonomic partitioning of *Cyrtodactylus louisiadensis* (Lacertilia: Gekkonidae) from Papua New Guinea. *Zootaxa* **2008,** *1883*, 1–27.

<https://doi.org/10.11646/zootaxa.1883.1.1>

57. Oliver, M.P.; Richards, S.J.; Mumpuni.; Rösler, H. The knight and the king: two new species of giant bent-toed gecko (*Cyrtodactylus,* Gekkonidae, Squamata) from northern New Guinea, with comments on endemism in North Papuan Mountains. *Zookeys* **2016,** *562*, 105–130.

<https://doi.org/10.3897/zookeys.562.6052>

58. Bauer, A.M.; Kunya, K.; Sumontha, M.; Niyomwan, P.; Panitvong, N.; Pauwels, O.S.G.; Chanhome, L.; Kunya, T. *Cyrtodactylus erythrops* (Squamata: Gekkonidae), a new cave-dwelling gecko from Mae Hong Son Province, Thailand. *Zootaxa* **2009,** *2124*, 51–62.

<https://doi.org/10.11646/zootaxa.2124.1.4>

59. Wood, Jr., P.L.; Grismer, L.L.; Muin, M.A.; Anuar, S.; Oaks, J.r.; Sites, Jr., J.W. A new potentially endangered limestone-associated Bent-toed Gecko of the *Cyrtodactylus pulchellus* (Squamata: Gekkonidae) complex from northern Peninsular Malaysia. *Zootaxa* **2020,** 4751, 437–460.

60. Husain, A.; Ray, P. First record of *Cyrtodactylus fasciolatus* (Blyth), the bent-toed banded gecko (Sauria: Gekkonidae: Gekkoninae) from Garhwal Hills. *J. Bombay Nat. Hist. Soc.* **1993,** *90*, 518.

61. Batuwita, S.; Bahir, M.M. Description of five new species of *Cyrtodactylus* (Reptilia: Gekkonidae) from Sri Lanka. *Raffles Bull. Zool.* **2005,** *12*, 351–380.

62. Luu, V.Q.; Dung, V.T.; Nguyen, T.Q.; Le, M.D.; Ziegler, T. A new species of the *Cyrtodactylus irregularis* complex (Squamata: Gekkonidae) from Gia Lai Province, Central Highlands of Vietnam. *Zootaxa* **2017,** *4362*, 385–404.

<https://doi.org/10.11646/zootaxa.4362.3.4>

63. Grismer, L.L.; Belabut, D.M.; Quah, E.S.H.; Chan, K.O.; Wood, P.L., Jr., Hasim, R. A new species of karst forest-adapted Bent-toed Gecko (genus *Cyrtodactylus* Gray, 1827) belonging to the *C. sworderi* complex from a threatened karst forest in Perak, Peninsular Malaysia. *Zootaxa* **2014,** *3755*, 434–446.

<https://doi.org/10.11646/zootaxa.3755.5.3>

64. Welton, L.J.; Siler, C.D.; Linkem, C.W.; Diesmos, A.C.; Brown, R.M. Philippine bent-toed geckos of the *Cyrtodactylus agusanensis* complex: multilocus phylogeny, morphological diversity, and descriptions of three new species. *Herpetol. Mon.* **2010,** *24*, 55–85.

<https://doi.org/10.1655/HERPMONOGRAPHS-D-10-00005.1>

65. Annandale, N. The Indian geckos of the genus *Gymnodactylus*. *Rec. Indian Mus.* **1913,** *9*, 309­­–326.

66. Grismer, L.L.; Wood Jr., P.L.; Anuar, S., Davis, H.R.; Cobos, A.J.; Murdoch, M.L. A new species of karst forest Bent-toed Gecko (genus *Cyrtodactylus* Gray) not yet threatened by foreign cement companies and a summary of Peninsular Malaysia’s endemic karst forest herpetofauna and the need for its conservation. *Zootaxa* **2016,** 4061, 1–17.

<https://doi.org/10.11646/zootaxa.4061.1.1>

67. Agarwal, I.; Mahony, S.; Giri, V.B., Chaitanya, R.; Bauer, A.M. Six new *Cyrtodactylus* (Squamata: Gekkonidae) from northeast India. *Zootaxa* **2018,** 4524, 501–535.

68.   Mirza, Z.A.; Bhosale, H.; Ansari, F.; Phansalkar, P.; Mandar; Sawant; Gowande, G.; Patel, H. A new species of geckos of the genus *Cyrtodactylus* Gray, 1827 from Arunachal Pradesh, India. *Evol. Syst.* In press.

69. Grismer, L.L.; Wood, J., P.L.; Anuar, S.; Grismer, M.S.; Quah, E.S.H.; Murdoch, M.L.; Muin, M.A.; Davis, H.R.; Aguliar, C.; Klabacka, R.; Cobos, A.J.; Aowphol, A.; Sites, Jr., J.R. Two new Bent-toed Geckos of the *Cyrtodactylus pulchellus* complex from Peninsular Malaysia and multiple instances of convergent adaptation to limestone forest ecosystems. *Zootaxa* **2016,** *4105*, 401–429.

<https://doi.org/10.11646/zootaxa.4105.5.1>

70. Duda, P.L.; Sahi, D.N. *Cyrtodactylus himalayanus*: A new gekkonid species from Jemmu, India. *J. Herpetol.* **1978,** *12*, 351–354.

<https://doi.org/10.2307/1563616>

71. Ngo, V.T.; Grismer, L.L.; Grismer, J.L. A new endemic cave dwelling species of *Cyrtodactylus* Gray, 1827 (Squamata: Gekkonidae) in Kien Giang Biosphere Reserve, Southwestern Vietnam. *Zootaxa* **2008,** *1967*, 53–62.

<https://doi.org/10.11646/zootaxa.1967.1.3>

72. Riedel, J.; Nordberg, E.; Schwarzkopf, L. Ecological niche and habitat use of an Australian gecko assemblage. *Israel Journal of Ecology & Evolution* **2020,** 66, 209–222.

<https://doi.org/10.1163/22244662-bja10002>

73. Schneider, N.; Luu, V.Q.; Sitthivong, S.; Teynié, A.; Le, M.D.; Nguyen, T.Q.; Ziegler, T. Two new species of *Cyrtodactylus* (Squamata: Gekkonidae) from northern Laos, including new finding and expanded diagnosis of *C. bansocensis.* *Zootaxa* **2020,** 4822: 503-530.
<https://doi.org/10.11646/zootaxa.4822.4.3>

74. Luu, V.Q.; Nguyen, T.Q.; Do, H.Q.; Ziegler, T. A new *Cyrtodactylus* (Squamata: Gekkonidae) from Huong Son limestone forest, Hanoi, northern Vietnam. *Zootaxa* **2011,** *3129*, 39–50.

<https://doi.org/10.11646/zootaxa.3129.1.3>

75. Ngo, V.T.; Bauer, A.M. Descriptions of two new species of *Cyrtodactylus* Gray, 1827 (Squamata: Gekkonidae) endemic to southern Vietnam. *Zootaxa* **2008,** *1715*, 27–42.

<https://doi.org/10.11646/zootaxa.1715.1.2>

76. Ulber, T. Bemerkungen über cyrtodactyline Geckos aus Thailand nebst Beschreibungen von zwei neuen Arten (Reptilia: Gekkonidae). *Mitt. Zool. Mus. Berlin* **1993,** *69*, 187–200.

<https://doi.org/10.1002/mmnz.19930690202>

77. Kunya, K.; Sumontha, M.; Panitvong, N.; Dongkumfu, W.; Sirisamphan, T.; Pauwels, O.S.G. A new forest-dwelling Bent-toed Gecko (Squamata: Gekkonidae: *Cyrtodactylus*) from Doi Inthanon, Chiang Mai Province, northern Thailand. *Zootaxa* **2015,** *3905*, 573–584.

<https://doi.org/10.11646/zootaxa.3905.4.9>

78. Nazarov, R.A.; Orlov, N.L.; Nguyen, N.C.; Ho, T. C. Taxonomy of naked-toes geckos *Cyrtodactylus irregularis* complex of south Vietnam and description of a new species from Chu Yang Sin Natural Park (Krong Bong district, Dac Lac province, Vietnam). *Russian J. Herpetol.*  **2008,** *15*, 141–156.

79. Luu, V.Q.; Calame, T.; Bonkowski, M.; Nguyen, T.Q.; Ziegler, T. A new species of *Cyrtodactylus* (Squamata, Gekkonidae) from Khammouane Province, Laos Zootaxa **2014,** *3760*, 54–66.

<https://doi.org/10.11646/zootaxa.3760.1.3>

80. Sumontha, M.; Kunya, K.; Pauwels, O.S.G. Jarujin’s bent toed gecko (*Cyrtodactylus jarujini*) in nature. *Ecol. notes.* *Rec. Natur.* **2008,** *2,* 22**–**23.

81. Grismer, L.L.; Wood, Jr., P.L.; Anuar, S.; Quah, E.S.H.; Muin, M.A.; Mohamed, M.; Chan, K.O.; Sumarli, A.x.; Loredo, A.I.; Heinz, H.M. The phylogenetic relationships of three new species of the *Cyrtodactylus pulchellus* complex (Squamata: Gekkonidae) from poorly explored regions in northeastern Peninsular Malaysia. *Zootaxa* **2014,** *3786*, 359–381.

<https://doi.org/10.11646/zootaxa.3786.3.6>

82. Hayden, C.J.; Brown, R.M.; Gillespie, G.; Setiadi, M.I.; Linkem, C.W.; Iskandar, D.T.; Umilaela, Bickford, D.P.; Riyanto, A.; Mumpuni, McGuire, J.A. A new species of bent-toed gecko *Cyrtodactylus* Gray, 1827, (Squamata: Gekkonidae) from the island of Sulawesi, Indonesia. *Herpetologica* **2008,** *64*, 109–120.

<https://doi.org/10.1655/07-026.1>

83. Li, P.-P. Description of a new subspecies of *Cyrtodactylus khasiensis* from China. *Acta Zootaxonom. Sin.* **2007,** *32*, 733–737.

<https://doi.org/10.1016/j.jss.2006.12.041>

84. Bauer, A. M. & Doughty, P. (2012). A new bent-toed gecko (Squamata: Gekkonidae: *Cyrtodactylus*) from the Kimberley region, Western Australia. *Zootaxa*, 3187, 32–42.

<https://doi.org/10.11646/zootaxa.3187.1.2>

85. Ziegler, T.; Phung, T.M.; Le, M.D.; Nguyen, T.Q. A new *Cyrtodactylus* (Squamata: Gekkonidae) from Phu Yen Province, southern Vietnam. *Zootaxa* **2013,** *3686*, 432–446.

<https://doi.org/10.11646/zootaxa.3686.4.2>

86. Grismer, L.L.; Chan, K.O.; Oaks, J.R.; Neang, T.; Lang, S.; Murdoch, M.L.; Stuart, B.L.; Grismer, J.L. A new insular species of the *Cyrtodactylus intermedius* (Squamata: Gekkonidae) group from Cambodia with a discussion of habitat preference and ecomorphology. *Zootaxa* **2020,** *4830*, 75–102. <https://doi.org/10.11646/zootaxa.4830.1.3>

87. Geissler, P.; Hartmann, T.; Ihlow, F.; Neang, T.; Rattanak, S.; Wagner, P.; Böhme, W. Herpetofauna of the Phnom Kulen National Park, northern Cambodia-an annotated checklist. *Cambodian J. Nat. Hist*. **2019,** *1,* 40–63.

88. Harvey, M.B.; O’connell, K.A.; Wostl, E.; Riyanto, A.; Kurniawan, N.; Smith, E.N.; Grismer, L.L. Redescription *Cyrtodactylus lateralis* (Werner) (Squamata: Gekkonidae) and Phylogeny of the Prehensile-tailed *Cyrtodactylus*. *Zootaxa*, **2016,** *4107*, 517–540.

<https://doi.org/10.11646/zootaxa.4107.4.3>

89. Chan, K.O.; Norhayati, A. A new insular species of *Cyrtodactylus* (Squamata: Gekkonidae) from northeastern Peninsula Malaysia, Malaysia. *Zootaxa*, **2010**, *2389*, 47–56.

<https://doi.org/10.11646/zootaxa.2389.1.2>

90. Connette, G.M.; Oswald, P.; Myint Kyaw Thura; Connette, K.J.L; Grindley, M.E.; Songer, M.; Zug, G.R.; Mulcahy, D.G. Rapid forest clearing in a Myanmar proposed national park threatens two newly discovered species of geckos (Gekkonidae: *Cyrtodactylus*). *PLoS One* **2017,***12*, *4*, e0174432.

<https://doi.org/10.1371/journal.pone.0174432>

91. Davis, H.R.; Bauer, A.M.; Jackman, T.R.; Nashriq, I.; Das, I. Uncovering karst endemism within Borneo: two new *Cyrtodactylus* species from Sarawak, Malaysia. *Zootaxa* **2019,** 4614, 331–352.

<https://doi.org/10.11646/zootaxa.4614.2.4>

92. Ngo, V.T.; Pauwels, O.S.G. A new cave-dwelling species of *Cyrtodactylus* Gray, 1827 (Squamata: Gekkonidae) from Khammouane province, southern Laos. *Zootaxa* **2010,** 2730, 44–56.

<https://doi.org/10.11646/zootaxa.2730.1.3>

93. dan Mulyadi, A.H. Herpetofauna of Waigeo Island (Herpetofauna di Pulau Waigeo). *Zool. Mus. Bogoriense, Indonesia* **2007,** Unpublished.

94. Grismer, L.L; Norhayati, A. A new insular species of *Cyrtodactylus* (Squamata: Gekkonidae) from the Langkawi Archipelago, Kedah, Peninsular Malaysia. *Zootaxa* **2008,** *1924*, 53–68.

<https://doi.org/10.11646/zootaxa.1924.1.3>

95. Grismer, L.L.; Rujirawan, A.; Termprayoon, K.; Ampai, N.; Yodthong, S.; Wood, Jr., P.L.; Oak, J.R.; Aowphol, A. A new species of *Cyrtodactylus* Gray (Squamata; Gekkonidae) from the Thai Highlands with a discussion on the evolution of habitat preference. *Zootaxa* **2020,** *4852*, 401–427. <https://doi.org/10.11646/zootaxa.4852.4.1>

96. Grismer, L.L.; Wood, Jr., P.L.; Lim, K.K.P. *Cyrtodactylus majulah*, a new species of bent-toed gecko (Reptilia: Squamata: Gekkonidae) from Singapore and the Riau Archipelago. *Raffles Bull. Zool.* **2012,** *60*, 487–499.

97. Davis, H.R.; Chan, K.O.; Das, I.; Brennan, I.G.; Karin, B.R.; Jackman, T.R.; Brown, R.F.; Iskandar, D.T.; Nashriq, I.; Grismer, L.L.; Bauer, A.M. Multilocus phylogeny of Bornean Bent-Toed geckos (Gekkonidae: *Cyrtodactylus*) reveals hidden diversity, taxonomic disarray, and novel biogeographic patterns. *Mol. Phylo. Evol.* **2020,** *147*, 1–11.

<https://doi.org/10.1016/j.ympev.2020.106785>

98. Grismer, L.L.; Wood, Jr., P.L.; Quah, E.S.H.; Murdoch, M.L.; Grismer, M.S.; Herr, M.W.; Espinoza, R.E.; Brown, R.M.; Aung Lin. A phylogenetic taxonomy of the *Cyrtodactylus peguensis* group (Reptilia: Squamata: Gekkonidae) with descriptions of two new species from Myanmar. *PeerJ*, **2018,** *6*, e5575.

<https://doi.org/10.7717/peerj.5575>

99. Grismer, L.L.; Wood, Jr., P.L.; Chan, K.O.; Anuar, S.; Muin, M.A. Cyrts in the city: a new Bent-toed Gecko (Genus *Cyrtodactylus*) is the only endemic species of vertebrate from Batu Caves, Selangor, Peninsular Malaysia. *Zootaxa* **2014,** *3774*, 381–394.

<https://doi.org/10.11646/zootaxa.3774.4.6>

100. Brown, W.C.; Parker, F. A new species of *Cyrtodactylus* (Gekkonidae) from New Guinea with a key to species from the island. *Breviora* **1973,** 417, 1–7.

101. Grismer, L.L.; Wood, Jr., P.L.; Quah, E.S.H.; Myint Kyaw Thura, Herr, M.W.; Aung Ko Lin. A new species of forest-dwelling *Cyrtodactylus* Gray (Squamata: Gekkonidae) from the Indawgyi Wildlife Sanctuary, Kachin State, Myanmar. *Zootaxa*, **2019,** *4623*, 1–25.

<https://doi.org/10.11646/zootaxa.4623.1.1>

102. Sitthivong, S.; Luu, V.Q.; Ha, N.V.; Nguyen, T.Q.; Le, D.M.; Ziegler. T. A new species of *Cyrtodactylus* (Squamata, Gekkonidae) from Vientiane Province, northern Laos. *Zootaxa* **2019,** *4701*, 257–275.

<https://doi.org/10.11646/zootaxa.4701.3.3>

103. Kraus, F.; Allison, A. A new species of *Cyrtodactylus* (Lacertilia: Gekkonidae) from Papua New Guinea. *Zootaxa* **2006,** *1247*, 59–68.

<https://doi.org/10.11646/zootaxa.1425.1.8>

104. Le, D.T.; Sitthivong, S.; Tran, T.T.; Grismer, L.L.; Nguyen, T.Q.; Le, M.D.; Ziegler, T.; Luu, V.Q. A new species of the *Cyrtodactylus brevipalmatus* complex (Squamata: Gekkonidae) from Dien Bien Province, northern Vietnam. *Zootaxa* in press.

105. Nguyen, S.N.; Orlov, N.L.; Darevsky, I.S. Descriptions of two new species of the genus *Cyrtodactylus* Gray, 1827 (Squamata: Sauria: Gekkonidae) from southern Vietnam. *J. Herpetol.* **2006,** *13*, 215–226.

106. Grismer, L.L.; Wood, Jr., P.L.; Myint Kyaw Thura, Nay Myo Win, Quah, E.S.H. Two more new species of the *Cyrtodactylus peguensis* group (Squamata: Gekkonidae) from the fringes of the Ayeyarwady Basin, Myanmar. *Zootaxa* **2019,** *4577*, 274–294.

<https://doi.org/10.11646/zootaxa.4577.2.3>

107. Nguyen, T.Q.; Le, M.D.; Pham, A.V.; Ngo, H.N.; Hoang, C.V.; Pham, C.T.; Ziegler, T. Two new species of *Cyrtodactylus* (Squamata: Gekkonidae) from the karst forest of Hoa Binh Province, Vietnam. *Zootaxa* **2015,** *3985*, 375–390.

<https://doi.org/10.11646/zootaxa.3985.3.3>

108. Schneider, N.; Nguyen, T.Q.; Schmitz, A.; Kingsada, P.; Auer, M.; Ziegler, T. A new species of karst dwelling *Cyrtodactylus* (Squamata: Gekkonidae) from northwestern Laos. *Zootaxa* **2011,** *2930*, 1–21.

<https://doi.org/10.11646/zootaxa.2930.1.1>

109. Johnson, C.B.; Quah, E.S.; Anuar, S.; Muin, M.A.; Wood, Jr., P.L.; Grismer, J.L.; Greer, L.F.; Chan, K.O.; Norhayati, A.; Bauer, A.M.; Grismer, L.L. Phylogeography, geographic variation and taxonomy of the bent-toed gecko *Cyrtodactylus quadrivirgatus* Taylor, 1962 from Peninsula Malaysia with the description of a new swamp dwelling species. *Zootaxa* **2012,** *3406*, 39–58.

<https://doi.org/10.11646/zootaxa.3406.1.3>

110. Riyanto, A.; Grismer, L.L.; Wood, Jr., P.L. The fourth Bent-toed Gecko of the genus *Cyrtodactylus* (Squamata: Gekkonidae) from Java, Indonesia *Zootaxa* **2015,** *4059*, 351–363.

<https://doi.org/10.11646/zootaxa.4059.2.6>

111. Brown, R.; Gaulke, M.; Rico, E. *Cyrtodactylus philippinicus*. The IUCN Red List of Threatened Species. Version 2014.2 **2009,** Available at: www.iucnredlist.org (accessed on 14 October 2014).

112. Neang, T.; Henson, A.; Stuart, B.L. A new species of *Cyrtodactylus* (Squamata, Gekkonidae) from Cambodia's Prey Lang Wildlife Sanctuary. *ZooKeys* **2020,** *926*, 133­158.
<https://doi.org/10.3897/zookeys.926.48671>

113. Loos, J.; von Wehdren, H.; Kien, N.D.; Ziegler, T. Niche segregation in microhabitat use of three sympatric *Cyrtodactylus* in the Phong Nha-Ke Bang National Park, central Vietnam. *Herpetol. Conserv. Biol.*  **2012,** *7*, 101–108.

114. Ostrowski, S.; Do, D.T.; Le, M.D.; Ngo, H.T.; Pham, C.T.; Nguyen, T.Q.; Nguyen, V.T.H.; Ziegler, T. A new species of *Cyrtodactylus* (Squamata: Gekkonidae) from southern Vietnam. *Zootaxa* **2020,** *4789*, 171–203.

<https://doi.org/10.11646/zootaxa.4789.1.5>

115. Nguyen, S.N.; Le, T.-N.T.; Tran, T.AD. Orlov, N.L.; Lathrop, A.M.Y.; Macculloch, R.D.; Le, T.-D.T.; Jin, J.-Q.; Nguyen, L.T.; Nguyen, T.T.; Hoang, D.D.; Che, J.; Murphy, R.W.; Zhang, Y.-P. Phylogeny of the *Cyrtodactylus irregularis* species complex (Squamata: Gekkonidae) from Vietnam with the description of two new species. *Zootaxa* **2013,** *3737*, 399–414.

<https://doi.org/10.11646/zootaxa.3737.4.4>

116. Ngo, V.T.; Grismer, L.L., Grismer, J.L. A new species of *Cyrtodactylus* Gray, 1827 (Squamata: Gekkonidae) in Phu Quoc National Park, Kien Giang Biosphere Reserve, southwestern Vietnam. *Zootaxa* **2010,** *2604*, 37–51.

<https://doi.org/10.11646/zootaxa.2604.1.3>

117. Grismer, L.L.; Wood, Jr., P.L.; Quah, E.S.H.; Myint Kyaw Thura, Oaks, J.R.; Aung Lin. A new species of Bent-toed Gecko (Squamata, Gekkonidae, *Cyrtodactylus*) from the Shan Plateau in eastern Myanmar (Burma). *Zootaxa* **2019,** *4624*, 301–321.

<https://doi.org/10.11646/zootaxa.4624.3.1>

118. Harvey, M.B.; O’connell, K.A.; Barraza, G.; Riyanto, A.; Kurniawan, N.; Smith, E.N. Two new species of *Cyrtodactylus* (Squamata: Gekkonidae) from the Southern Bukit Barisan Range of Sumatra and an estimation of their phylogeny. *Zootaxa* **2015,** *4020,* 495–516.

<https://doi.org/10.11646/zootaxa.4020.3.5>

119. Rösler, H.; Thanh, V.N.; Truong, N.Q.; Tri, N.V.; Ziegler, T. A new *Cyrtodactylus* (Squamata: Gekkonidae) from Central Vietnam. *Hamadryad* **2008,** *33*, 48–63.

120. Nguyen, S.N.; Yang, J.-X.; Le, T.-N.T.; Nguyen, L.T.; Orlov, N.L.; Hoang, C.V.; Nguyen, T.Q.; Jin, J.-Q.; Rao, D.-Q.; Hoang, T.N.; Che, J.; Murphy, R.W.; Zhang, Y-P. DNA barcoding of Vietnamese bent-toed geckos (Squamata: Gekkonidae: *Cyrtodactylus*) and the description of a new species. *Zootaxa* **2014,** *3784*, 48–66.

<https://doi.org/10.11646/zootaxa.3784.1.2>

121. Nguyen, A.T.; Duong, T.V.; Grismer, L.L.; Poyarkov, N.A. A new granite cave-dwelling Bent-toed Gecko from Vietnam of the *Cyrtodactylus irregularis* group (Squamata; Gekkonidae) and a discussion on cave ecomorphology *Vert. Zool.* in press

122. Brown, R.; Dimalibot, J. *Cyrtodactylus redimiculus.* The IUCN Red List of Threatened Species. **2009,** Version 2014.2 Available at: www.iucnredlist.org (accessed on 14 October 2014).

123. Agarwal, I. Two new species of ground-dwelling *Cyrtodactylus* (*Geckoella*) from the Mysore Plateau, south India. *Zootaxa* **2016,** 4193, 228–244.

<https://doi.org/10.11646/zootaxa.4193.2.2>

124. Ziegler, T.; Nazarov, R.; Orlov, N.; Nguyen, T.Q.; Vu, T.N.; Dang, K.N.; Dinh, T.H.; Schmitz, A. A third new *Cyrtodactylus* (Squamata: Gekkonidae) from Phong Nha-Ke Bang National Park, Truong Son Range, Vietnam. *Zootaxa* **2010,** *2413*, 20–36.

<https://doi.org/10.11646/zootaxa.2413.1.2>

125. Chandramouli, S.R. A review of the gekkonid genus *Cyrtodactylus* Gray, 1827 (Sauria: Gekkonidae) in the Andaman and Nicobar archipelago with the description of two new species from the Nicobar Islands. *Asian J. Conserv. Biol.* **2020,** 9, 78–89.

126. Smith, M.J.; Cogger, H.; Tiernan, B.; Maple, D.; Boland, C.; Napier, F.; Detto, T., Smith, P. An oceanic island reptile community under threat: the decline of reptiles on Christmas Island, Indian Ocean. *Herpetol. Conserv. Biol.* **2012,** *7*, 206–218.

127. Aksornneam, A.; Sung, Y.-H.; Aowphol, A. Microhabitat use of two sympatric geckos *Cyrtodactylus* in limestone forest, Western Thailand. Abstract for AVIS conference, Hanoi, Vietnam, 2019.

128. Panitvong, N.; Sumontha, M.; Tunprasert, J.; Pauwels, O.S.G. *Cyrtodactylus saiyok* sp. nov., a new dry evergreen forest-dwelling Bent-toed Gecko (Squamata: Gekkonidae) from Kanchanaburi Province, western Thailand. *Zootaxa* **2014**, *3869*, 64–74.

<https://doi.org/10.11646/zootaxa.3869.1.6>

129. Rösler, H.; Richards, S.J.; Günther, R. Remarks on morphology and taxonomy of geckos of the genus *Cyrtodactylus* Gray, 1827, occurring east of Wallacea, with descriptions of two new species (Reptilia: Sauria: Gekkonidae). *Salamandra* **2007,** *43*, 193–230.

130. Pauwels, O.S.G.; Nazarov, R.A.; Bobrov, V.V.; Poyarkov, N.A. Taxonomic status of two populations of Bent-toed Geckos of the *Cyrtodactylus irregularis* complex (Squamata, Gekkonidae) with description of a new species from Nui Chua National Park, southern Vietnam. *Zootaxa* **2018,** *4403*, 307-335. <https://doi.org/10.11646/zootaxa.4403.2.5>

131. Pauwels, O.S.G.; Sumontha, M.; Latinne, A.; Grismer, L.L. *Cyrtodactylus sanook* (Squamata: Gekkonidae), a new cave-dwelling gecko from Chumphon Province, southern Thailand. *Zootaxa* **2013,** *3635*, 275–285.

<https://doi.org/10.11646/zootaxa.3635.3.7>

132. Grismer, L.L.; Leong, T.M. New species of *Cyrtodactylus* (Squamata: Gekkonidae) from Southern Peninsular Malaysia. *J. Herpetol.* **2005,** *39*, 584–591.

<https://doi.org/10.1670/43-05A.1>

133. Youmans, T.M.; Grismer, L.L. A new species of *Cyrtodactylus* (Reptilia: Squamata: Gekkonidae) from the Seribuat Archipelago, West Malaysia *Herpetol. Nat.l Hist.* **2006,** *10*, 61–70.

134. Luu, V.Q.; Bonkowski, M.; Nguyen, T.Q.; Le, M.D.; Schneider, N.; Ngo, H.T.; Ziegler, T. Evolution in karst massifs, cryptic diversity among bent-toed geckos along the Truong Son Range with descriptions of three new species and one new country record from Laos. *Zootaxa* **2016,** *4107*, 101–140. <https://doi.org/10.11646/zootaxa.4107.2.1>

135. Le, D.T.; Nguyen, T.Q.; Le, M.D.; Ziegler, T. A new species of *Cyrtodactylus* (Squamata: Gekkonidae) from Ninh Binh Province, Vietnam. *Zootaxa* **2016,** *4162*, 268–282.

<https://doi.org/10.11646/zootaxa.4162.2.4>

136. Nguyen, T.Q.; Pham, A.V.; Ziegler, T.; Ngo, T.H.; & Le, M.D. A new species of *Cyrtodactylus* (Squamata: Gekkonidae) and the first record of *C. otai* from Son La Province, Vietnam. *Zootaxa* **2017,** *4341*, 25–40.

<https://doi.org/10.11646/zootaxa.4341.1.2>

137. Luu, V.Q.; Calame, T.; Nguyen, T.Q.; Bonkowski, M.; Ziegler, T. A new species of *Cyrtodactylus* (Squamata: Gekkonidae) from the limestone forest of Khammouane Province, central Laos. *Zootaxa* **2015,** *4058*, 388–402.

<https://doi.org/10.11646/zootaxa.4058.3.6>

138. Nielsen, S.V.; Oliver, P.M. Morphological and genetic evidence for a new karst specialist lizard from New Guinea (*Cyrtodactylus*: Gekkonidae) *Royal Soc. Open Sci.,* **2017,** *4*, 170781.

<https://doi.org/10.1098/rsos.170781>

139. Linkem, C.W.; McGuire, J.A.; Hayden, C.J.; Setiadi, M.I.; Bickford, D.P.; Brown, R.M. A new species of bent-toed gecko (Gekkonidae: *Cyrtodactylus*) from Sulawesi Island, Indonesia. *Herpetologica* **2008,** *64*, 224–234.

<https://doi.org/10.1655/07-040.1>

140. Pham, A.V.; Le, M.D.; Ziegler, T.; Nguyen, T.Q. A new species of *Cyrtodactylus* (Squamata: Gekkonidae) from northwestern Vietnam. *Zootaxa* **2019,** *4544*, 360–380.

<https://doi.org/10.11646/zootaxa.4544.3.3>

141. Grismer, L.L.; Anuar, S.; Muin, M.A.; Quah, E.S.H.; Wood, Jr., P.L. Phylogenetic relationships and description of a new upland species of Bent-toed Gecko (*Cyrtodactylus* Gray, 1827) of the *C. sworderi* complex from northeastern Peninsular Malaysia. *Zootaxa* **2013,** *3616*, 239–52.

<https://doi.org/10.11646/zootaxa.3616.3.2>

142. David, P.; Nguyen, T.Q.; Schneider, N.; Ziegler, T. A new species of *Cyrtodactylus* Gray, 1827 from central Laos (Squamata: Gekkonidae). *Zootaxa* **2011,** *2833*, 29–40.

<https://doi.org/10.11646/zootaxa.2833.1.3>

143. Pauwels, O.S.G.; Bauer, A.M.; Sumontha, M.; Chanhome, L. *Cyrtodactylus thirakhupti* (Squamata: Gekkonidae), a new cave-dwelling gecko from southern Thailand. *Zootaxa* **2004,** *772*, 1–11.

<https://doi.org/10.11646/zootaxa.772.1.1>

144. Shi, L.; Zhao, H. A new species of *Cyrtodactylus* (Reptilia: Squamata: Gekkonidae) from Xizang Autonomous Region, China. *Zootaxa* **2010,** *2336*, 51–60.

<https://doi.org/10.11646/zootaxa.2336.1.4>

145. Grismer, J.L.; Grismer, L.L.; Das, I.; Yaakob, N.S. Lim, L.B.; Tzi, M.L.; Youmans, T.M.; Kaiser, H. Species diversity and checklist of the herpetofauna of Pulau Tioman, Peninsular Malaysia, with a preliminary overview of habitat utilization. *Asiatic Herpetol. Res.* **2004,** *10*, 247–279.

146. Botejue, W.M.S.; Wattavidanage, J. Herpetofaunal diversity and distribution in Kalugala proposed forest reserve, Western province of Sri Lanka. *Amphib. Rept. Conserv.* **2012,** *5*, 65–80.

147. Worthington, J.; Wilmer, A.B.; Couper, P. Phylogeography of north-eastern Australia’s *Cyrtodactylus* radiation: a habitat switch highlights adaptive capacity at a generic level. *Australian J. Zool.* **2015,** *63,* 398–410.

<https://doi.org/10.1071/ZO15051>

148. Purkayastha, J.; Das, M.; Bohra, S.C.; Bauer, A.M.; Agarwal, I. Another new *Cyrtodactylus* (Squamata: Gekkonidae) from Guwahati, Assam, India. *Zootaxa* **2020,** 4732, 375­392.

<https://doi.org/10.11646/zootaxa.4732.3.2>

149. Agarwal, I.; Mirza, Z.A.; Pal, S.; Maddock, S.T.; Mishra, A.; Bauer, A.M. A new species of the *Cyrtodactylus* (*Geckoella*) *collegalensis* (Beddome, 1870) complex (Squamata: Gekkonidae) from Western India. *Zootaxa* **2016,** 4170, 339–354.

150. Nguyen, T.Q.; Kingsada, P.; Rösler, H.; Auer, M.; Ziegler, T. A new species of *Cyrtodactylus* (Squamata: Gekkonidae) from northern Laos. *Zootaxa* **2010,** *2652*, 1–16.

<https://doi.org/10.11646/zootaxa.2652.1.1>

151. Yuan, S.Q.; Rao, D. Q. A new record of a gekkonid (*Cyrtodactylus wayakonei*) from Yunnan, China. *Zool. Res.* **2011,** *32*, 684–688.

152. Deraniyagala, P.E.P. A new gymnodactylid gecko from Ceylon. *Spol. Zeylan.* **1945,** *24*, 99–102.

153 Ngo, V.T.; Chan, K.O. A new species of *Cyrtodactylus* Gray, 1826 (Squamata: Gekkonidae) from Khanh Hoa province, Southern Vietnam. *Zootaxa*, **2010,** *2504*, 47–60.

154. Grismer, L.L.; Wood, Jr., P.L.; Myint Kyaw Thura, Quah, E.S.H.; Grismer, M.S.; Murdoch, M.L.; Espinoza, R.E.; Aung Lin. A new *Cyrtodactylus* Gray, 1827 (Squamata, Gekkonidae) from the Shan Hills and the biogeography of Bent-toed Geckos from eastern Myanmar. *Zootaxa* **2018,** 4446, 477–500.

<https://doi.org/10.11646/zootaxa.4446.4.4>

155. Taylor, E.H. The lizards of Thailand. *Univ. Kansas Sci. Bull.* **1963,** *44*, 687–1077.

156. Liu, S.; Rao, D. A new species of *Cyrtodactylus* Gray, 1827 (Squamata: Gekkonidae) from Yunnan, China. *Zootaxa* **2021,** in press.

157. Oliver, P.; Tjaturadi; Mumpuni; Krey, K.; Richards, S.J. A new species of large *Cyrtodactylus* (Squamata: Gekkonidae) from Melanesia. *Zootaxa* **2008,** *1894*, 59–68.
<https://doi.org/10.11646/zootaxa.1894.1.5>
